# Donor impurity-related linear and nonlinear intraband optical absorption coefficients in quantum ring: effects of applied electric field and hydrostatic pressure

**DOI:** 10.1186/1556-276X-7-538

**Published:** 2012-09-28

**Authors:** Manuk G Barseghyan, Ricardo L Restrepo, Miguel E Mora-Ramos, Albert A Kirakosyan, Carlos A Duque

**Affiliations:** 1 Department of Solid State Physics, Yerevan State University, Al. Manookian 1, Yerevan, 0025, Armenia; 2 Escuela de Ingeniería de Antioquia, Medellín, 7516, Colombia; 3 Morelos State University, Cuernavaca, 62209, Morelos, Mexico; 4 Instituto de Física, Universidad de Antioquia, Medellín, 1226, Colombia

**Keywords:** GaAs, Quantum ring, Optical absorption, 78.67.De; 71.55.Eq; 32.10.Dk

## Abstract

The linear and nonlinear intraband optical absorption coefficients in *GaAs* three-dimensional single quantum rings are investigated. Taking into account the combined effects of hydrostatic pressure and electric field, applied along the growth direction of the heterostructure, the energies of the ground and first excited states of a donor impurity have been found using the effective mass approximation and a variational method. The energies of these states are examined as functions of the dimensions of the structure, electric field, and hydrostatic pressure. We have also investigated the dependencies of the linear, nonlinear, and total optical absorption coefficients as a function of incident photon energy for several configurations of the system. It is found that the variation of distinct sizes of the structure leads to either a redshift and/or a blueshift of the resonant peaks of the intraband optical spectrum. In addition, we have found that the application of an electric field leads to a redshift, whereas the influence of hydrostatic pressure leads to a blueshift (in the case of on-ring-center donor impurity position) of the resonant peaks of the intraband optical spectrum.

## Background

The nonlinear optical properties of low-dimensional semiconductor systems such as quantum wells (QWs), quantum dots (QDs), quantum rings (QRs), and other nanostructures have attracted much attention in some areas of applied physics [1-4]. The reason is that the nonlinear optical properties typical of the low-dimensional materials have great potential for device applications in laser amplifiers [5], photodetectors [1], high-speed electro-optical modulators [2], and so on.

On other hand, the investigation of the electronic properties of hydrogen-like impurities in low-dimensional semiconductor heterostructures also attracts pretty much interest. It is explained by the vast possibility of purposeful manipulation of the impurity binding energy by means of external influences and, hence, the possibility of controlling the electronic and optical properties of functional devices based on such heterostructures [6].

In accordance with both fundamental and applied researches, the simultaneous effect of impurity and external influences on the linear and nonlinear optical properties of semiconductor nanostructures has attracted much attention in recent years [7-18].

To our knowledge, there are only a few research articles related with the effects of impurity and external influences on linear and nonlinear optical properties of QRs [11,19]. The linear and the third-order nonlinear optical absorption spectra of a donor impurity confined within a QR with a parabolic potential have been investigated in [11]. Calculations are performed with the use of the matrix diagonalization method and the compact density matrix approach in the frame of the effective mass approximation. The authors have found that the modifications in the confinement strength, the incident optical density, and the ring radius have a great effect on the linear, the third-order nonlinear, and total absorption spectra. Furthermore, the second-order nonlinear optical rectification coefficient associated with intersubband transitions in a hydrogenic QR system with a two-dimensional pseudopotential in the presence of an external magnetic field is theoretically investigated in [19]. In that case, the calculations are performed using the perturbation method and the compact effective mass density matrix approach. According to the results, the second-order nonlinear optical rectification coefficient of a hydrogenic QR is strongly affected by the geometrical size and chemical potential of the pseudopential, the hydrogenic impurity, and the external magnetic field.

In the present work, the effects of hydrostatic pressure, in-growth-direction applied electric field, as well as the changes of the different dimensions of the structure’s geometry on the linear and the nonlinear intraband optical transitions in GaAs three-dimensional cylindrical QRs, are investigated. The paper is organized as follows: In the ‘Theoretical framework’ section, we describe the theoretical framework. The ‘Results and discussion’ section is dedicated to the results and discussion, and our conclusions are given in the ‘Conclusions’ section.

## Methods

### Theoretical framework

The Hamiltonian of the electron in the *GaAs* QR within the effective mass and parabolic band approximations, taking into account the influence of an in-growth-direction applied electric field, is given by the expression

(1) $$\begin{array}{c} H=-\frac{\hbar^{2}}{2\;m^{*}(P,T)}\left[\frac{1}{\rho }\frac{\partial }{\partial \rho }\left(\rho \frac{\partial }{\partial \rho }\right)+\frac{1}{\rho^{2}}\frac{\partial^{2}}{\partial \phi^{2}}+\frac{\partial^{2}}{\partial z^{2}}\right]\\ \;+V(\rho ,z,P)+|e|\;F\;z-\frac{e^{2}}{\varepsilon (P,T)\;r}\;, \end{array}$$

where $r={\left[{\left(\vec{\rho }-\vec{\rho_{i}}\right)}^{2}+{\left(z-z_{i}\right)}^{2}\right]}^{\frac{1}{2}}$ is the distance from the electron to the impurity site (with $\left(z_{i},\vec{\rho_{i}}\right)$ and $\left(z,\vec{\rho }\right)$ being the impurity and electron coordinates, respectively). Besides, *F* labels the strength of the dc electric field, while *e* is the absolute value of the electron charge. Additionally, *m*^∗^(*P*,*T*) and *ε*(*P*,*T*) are, respectively, the hydrostatic pressure- and temperature-dependent (*T* = 4 K in this work) electron effective mass and the static dielectric constant. *V*(*ρ*,*z*,*P*) = *V* (*z*,*P*) + *V* (*ρ*,*P*) is the confinement potential of the QR, given by

(2) $$V(\rho ,z,P)=\left\{\begin{array}{c} 0,\;\;\;\mathrm{if}\;R_{1}(P)\leq \rho \leq R_{2}(P),|z|<L(P)/2\\ \infty ,\;\;\text{if otherwise .} \end{array}\right)\;$$

To describe the effect of the impurity, the variational method shall be used. In the present work, we are strictly interested in the ground (1*s*) and first excited (2*s*) states of the confined electron. Therefore, the trial functions for the mentioned impurity states can be written as the products between the uncorrelated first confined subband eigenfunctions - associated with the electron motion in the QR - and a 1*s*- and a 2*s*-like hydrogenic functions of spherical character, respectively [20-24]. We have chosen the following functions as the wave functions of 1*s* and 2*s* states:

(3) $$\Psi_{i}(\rho ,z)=N_{i}\vartheta (\rho )f(z)e^{-\alpha_{i}r}$$

(4) $$\Psi_{f}(\rho ,z)=N_{f}\vartheta (\rho )f(z)(1-\beta_{f})e^{-\alpha_{f}r},$$

where *N*_*i*_ and *N*_*f*_ are the normalization constants, and {*α*_*i*_,*β*_*f*_,*α*_*f*_} are the variational parameters, which can be determined by also requiring the *Ψ*_*i*_ and *Ψ*_*f*_ forms of the set of orthogonal functions. The ground state wave functions *ϑ*(*ρ*) and *f(z)* without the impurity potential have the following forms [25,26]:

(5) $$\vartheta (\rho )=J_{0}\left(k\;\rho \right)+G_{1}Y_{0}(k\;\rho )$$

(6) $$f(z)=\text{Ai}(Z)+G_{2}\text{Bi}(Z),$$

where (*J*_0_*Y*_0_) are the first- and second-kind Bessel functions of order zero, respectively; $k={\left(\frac{2m^{*}(P,T)}{\hbar^{2}}E_{\rho }\right)}^{1/2}$ (*E*_*ρ*_ is the ground-state energy associated with the lateral confinement). On the other hand, (*Ai,Bi*) are the Airy functions, and *G*_1_ and *G*_2_ are the constants obtained from the continuity condition of the solutions at the interfaces; *Z* = [2*m*^∗^(*PT*)*eF* / *ℏ*^2^]^1/3^[*z*−*E*_*z*_/(*eF*)] (*E*_*z*_is the ground-state energy associated with the perpendicular confinement). For the 1*s*-like state, the variational procedure involves minimizing 〈*Ψ*_*i*_|*H*|*Ψ*_*i*_〉with respect to *α*_1*s*_ in order to find the impurity ground-state energy *E*_1*s*_. For the excited 2*s*-like state, a similar procedure is followed. The inclusion of hydrostatic pressure effects is made via the pressure dependence on the electron effective mass, the GaAs static dielectric constant, and on the dimensions (inner and outer radii and height of the heterostructure). They are respectively given by [26,27]

(7) $$\begin{array}{c} m^{*}(P,T)\;=\;{\left[1+\frac{15020\;\text{meV}}{E_{g}(P,T)}+\frac{7510\;\text{meV}}{E_{g}(P,T)+341\;\text{meV}}\right]}^{-1}\;m_{0}, \end{array}$$

(8) $$\begin{array}{c} \varepsilon (P,T)=12.74\times \text{exp}(-1.67\times 10^{-3}\;{\text{kbar}}^{-1}P)\\ \;\times \text{exp}\left[9.4\times 10^{-5}\;{\text{K}}^{-1}(T-75.6\;\text{K})\right], \end{array}$$

(9) $$L(P)=L(0)[1-P(S_{11}+2S_{12})]\;,$$

and

(10) $$R_{i}(P)=R_{i}(0){\left[1-2P(S_{11}+2S_{12})\right]}^{1/2},(i=1,2)\;,$$

where *m*_0_ is the free electron mass, and *E*_*g*_(*PT*) is the pressure- and temperature-dependent GaAs bandgap, determined by the following relation:

(11) $$\begin{array}{c} E_{g}(P,T)=\left(1519+10.7\;{\text{kbar}}^{-1}\;P-\frac{0.5405\;{\text{K}}^{-1}\;T^{2}}{T+204\;\text{K}}\right)\;\text{meV}. \end{array}$$

In the calculations, the values *S*_11_ = 1.16×10^−3^ kbar^−1^and *S*_12_ = −3.7×10^−4^ kbar^−1^ are taken.

With use of the density matrix approach, the linear and third-order optical absorption coefficients can be written, respectively, as [28,29]

(12) $$\alpha^{\left(1\right)}(\hbar \omega )=\frac{4\;\Pi \;\omega \;e^{2}}{\varepsilon {\left(P,T\right)}^{1/2}\;c}\frac{\sigma_{s}{\left|M_{\text{fi}}\right|}^{2}\Gamma_{0}}{{\left(E_{\text{fi}}-\hbar \omega \right)}^{2}+\Gamma_{0}^{2}}$$

and

(13) $$\begin{array}{lll} \alpha^{\left(3\right)}(\hbar \omega ,I) & =-\frac{32\;\Pi^{2}\;\omega \;e^{4}}{\varepsilon (P,T)\;c^{2}}\frac{I\;\sigma_{s}\;{\left|M_{\text{fi}}\right|}^{4}\;\Gamma_{0}}{{\left[\left(\right)E_{\text{fi}}\;-\;\hbar \omega {))}^{2}\;+\;\Gamma_{0}^{2}\right]}^{2}}\;\left[\;1\;-\;{\left|\frac{M_{\text{ff}}\;-\;M_{\text{ii}}}{2M_{\text{fi}}}\right|}^{2}\right)\; & \;\\ & \;\times \left(\frac{\left(\right)E_{\text{fi}}-\hbar \omega {))}^{2}-{\left(\Gamma_{0}\right)}^{2}+2E_{\text{fi}}\left(\right)E_{\text{fi}}-\hbar \omega ))}{E_{\text{fi}}^{2}+\Gamma_{0}^{2}}\right]\;,\; \end{array}$$

where *Γ*_0_( = 0.4 meV) is the Lorentzian - damping-related - parameter.

In expressions (12) and (13), the intensity of the incident field is labeled by *I*; *σ*_*s*_( = 3×10^16^cm^−3^) is the density of the electrons in the system, *E*_*fi*_ = *E*_*f*_−*E*_*i*_, and *M*_*fi*_ = 〈*Ψ*_*f*_| *ρ* cos (*ϕ*)|*Ψ*_*i*_〉 is the matrix element of the dipole operator.

## Results and discussion

**Figure 1:**
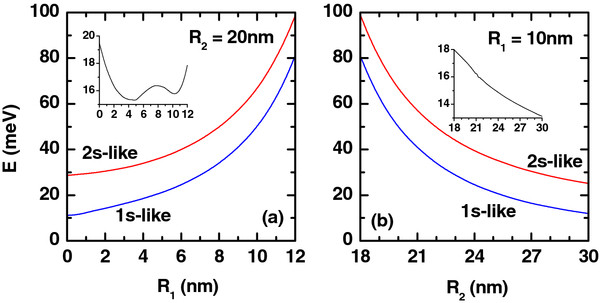
**Absorption coefficient (colored lines) as a function of photon energy.** Solid lines are for *α*_1_(*ℏω*). Dashed lines are for *α*_3_(*ℏω*,*I*), and dotted lines are for *α*(*ℏω*,*I*) = *α*_1_(*ℏω*) + *α*_3_(*ℏω*,*I*). The results are for *R*_2_ = 20 nm, *L* = 20 nm, *P* = 0, *F* = 0, and *I* = 2×10^4^W/cm^2^. Several values of the inner radius have been considered.

**Figure 2:**
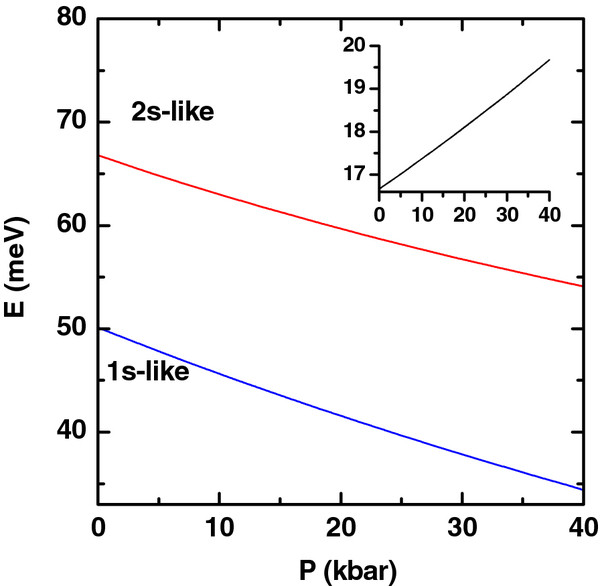
**Absorption coefficient (colored lines) as a function of photon energy.** Solid lines are for *α*_1_(*ℏω*). Dashed lines are for $\alpha_{3}(\hbar \omega ,I)$, and dotted lines are for *α*(*ℏω*,*I*) = *α*_1_(*ℏω*) + *α*_3_(*ℏω*,*I*). The results are for *R*_1_ = 10 nm, *L* = 20 nm, *P* = 0, *F* = 0, and *I* = 2×10^4^W/cm^2^. Several values of the outer radius have been considered.

**Figure 3:**
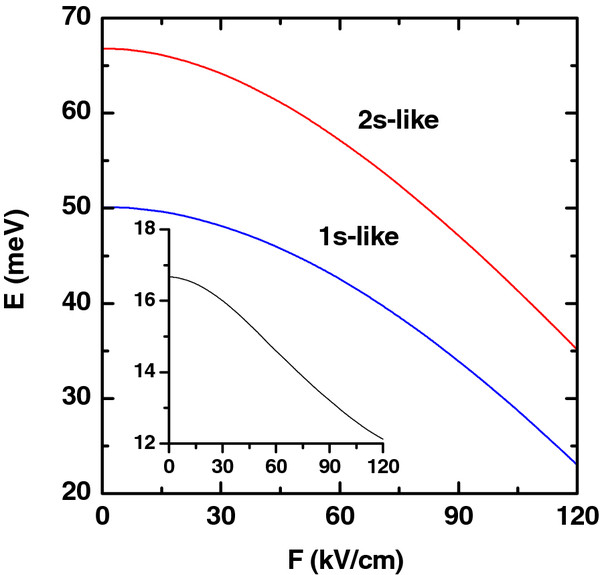
**Absorption coefficient (colored lines) as a function of photon energy.** Solid lines are for *α*_1_(*ℏω*). Dashed lines are for *α*_3_(*ℏω*,*I*), and dotted lines are for *α*(*ℏω*,*I*) = *α*_1_(*ℏω*) + *α*_3_(*ℏω*,*I*). The results are for *R*_1_ = 10 nm, *R*_2_ = 20 nm, *L* = 20 nm, *F* = 0, and *I* = 2×10^4^W/cm^2^. Several values of the hydrostatic pressure have been considered.

**Figure 4:**
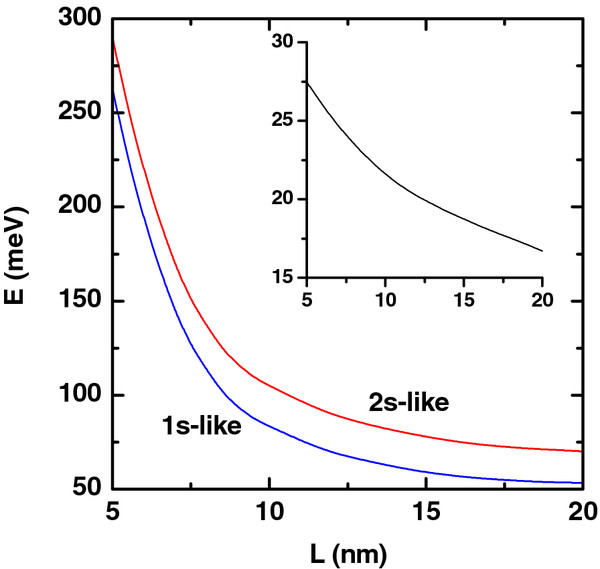
**Absorption coefficient (colored lines) as a function of photon energy.** Solid lines are for *α*_1_(*ℏω*). Dashed lines are for *α*_3_(*ℏω*,*I*), and dotted lines are for *α*(*ℏω*,*I*) = *α*_1_(*ℏω*) + *α*_3_(*ℏω*,*I*). The results are for *R*_1_ = 10 nm, *R*_2_ = 20 nm, *L* = 20 nm, *P* = 0, and *I* = 2×10^4^W/cm^2^. Several values of the applied electric field have been considered.

**Figure 5:**
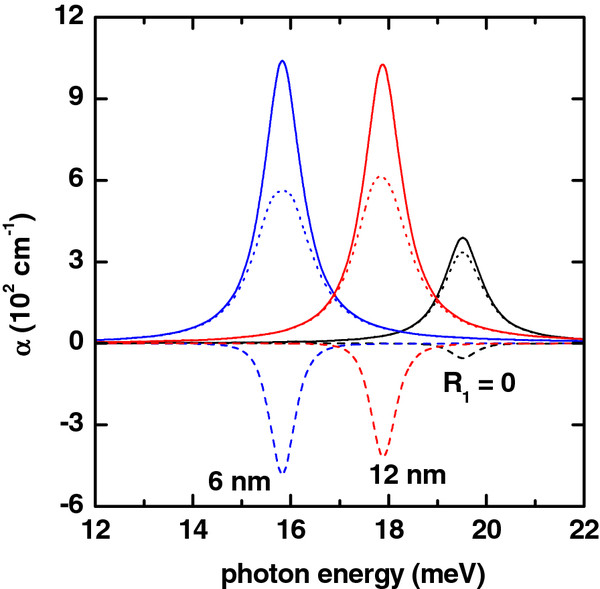
**Absorption coefficient (colored lines) as a function of photon energy.** Solid lines are for *α*_1_(*ℏω*). Dashed lines are for *α*_3_(*ℏω*,*I*), and dotted lines are for *α*(*ℏω*,*I*) = *α*_1_(*ℏω*) + *α*_3_(*ℏω*,*I*). The results are for *R*_1_ = 10 nm, *R*_2_ = 20 nm, *F* = 0, *P* = 0, and *I* = 2×10^4^W/cm^2^. Several values of the height of the ring have been considered.

**Figure 6:**
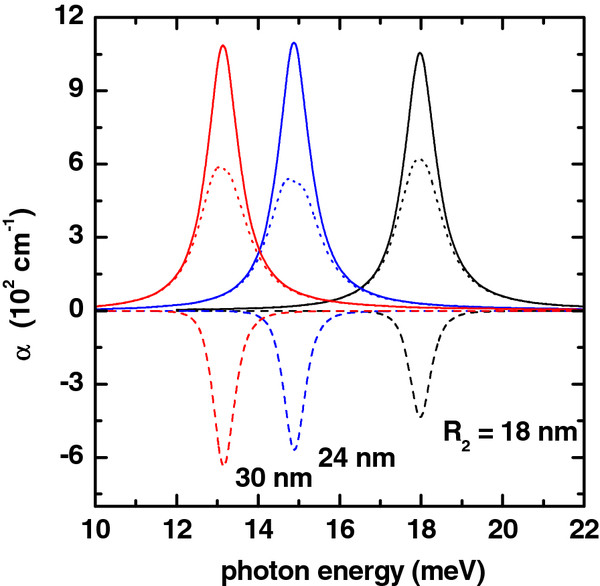
**Ground and first excited states.** Ground and first excited state energies of the electron (colored lines) as functions of the (**a**) inner and (**b**) outer radii of the QR for *L*=20 nm, *P*=0, and *F*=0. The impurity is placed at *z*_*i*_ = 0 and *ρ*_*i*_ =  (*R*_1_ + *R*_2_)/2. The insets show the corresponding energy difference between the 2*s* and 1*s* states.

**Figure 7:**
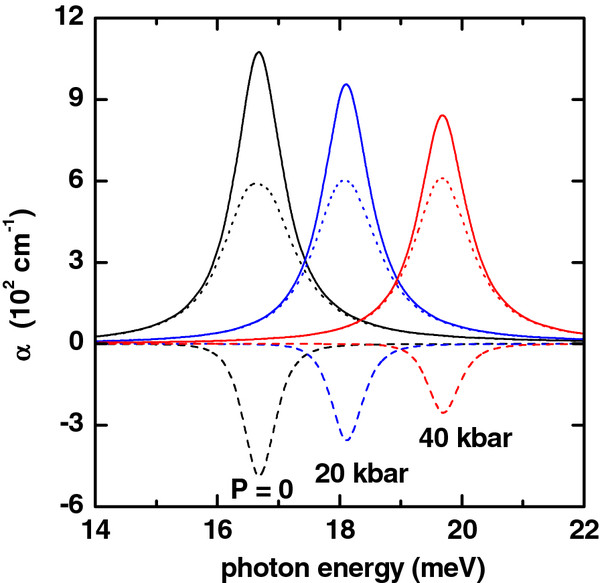
**Ground and first excited states.** Ground and first excited state energies of the electron (colored lines) as functions of hydrostatic pressure. The calculations are for *R*_1_ = 10 nm, *R*_2_ = 20 nm, *L* = 20 nm, and *F* = 0. The impurity is placed at *z*_*i*_ = 0 and *ρ*_*i*_ = (*R*_1_ + *R*_2_)/2. The inset shows the corresponding energy difference between the 2*s* and 1*s* states.

**Figure 8:**
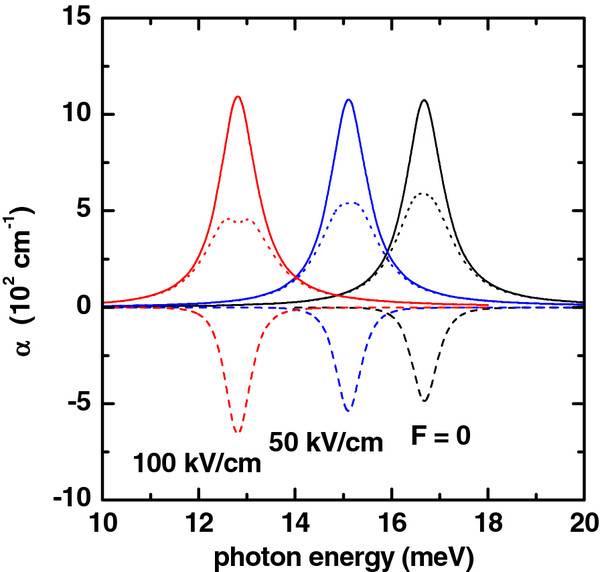
**Ground and first excited states.** Ground and first excited state energies of the electron (colored lines) as functions of the applied electric field. The calculations are for *R*_1_ = 10 nm, *R*_2_ = 20 nm, *L* = 20 nm, and *P* = 0. The impurity is placed at *z*_*i*_ = 0 and *ρ*_*i*_ = (*R*_1_ + *R*_2_)/2. The inset shows the corresponding energy difference between the 2*s* and 1*s* states.

**Figure 9:**
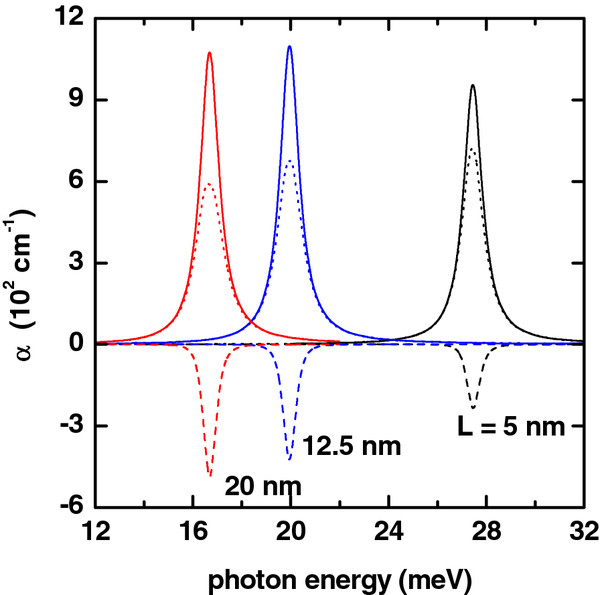
**Ground and first excited states.** Ground and first excited state energies of the electron (colored lines) as functions of the height of the QR *L*. The calculations are for *R*_1_ = 10 nm, *R*_2_ = 20 nm, *F* = 0, and *P* = 0. The impurity is placed at *z*_*i*_ = 0 and *ρ*_*i*_ = (*R*_1_ + *R*_2_)/2. The inset shows the corresponding energy difference between the 2*s* and 1*s* states.

In this section, we present the outcome of our calculations for the impurity energy levels and the associated optical properties of interest. For the sake of illustration, the figures containing the results on the energies also show - as insets - the corresponding 2*s*-1*s* energy differences, which associate with the resonant peak positions of the absorption coefficients. Then, the discussion regarding the results in Figures 1, 2, 3, 4, and 5 will make use of those contained in the insets of Figures 6, 7, 8, and 9.

Our results for the ground and first excited state energies of a donor impurity in a GaAs QR are shown in Figure 6a,b as functions of the inner and outer radii, respectively. For a fixed value of the outer radius (Figure 6a), the layer thickness *W* = *R*_2_−*R*_1_decreases as long as the inner radius is augmented with the consequent increment in the size quantization. As a result of this, there will be an increase of both the ground and first excited state energies. Moreover, a growth in the value of the ring’s outer radius is reflected in the decrease of the energies because of the weakening of the size quantization effect (see Figure 6b). As can be seen from Figure 6a,b, the influence of size quantization is much stronger for the excited states. This fact was expected because the excited state is more spread out inside the ring region than the ground state.

The results regarding the effect of hydrostatic pressure on the 1*s* and 2*s* state energies of a donor impurity in a GaAs QR can be found in Figure 7. It is clear that in all cases, the influence of the hydrostatic pressure has the effect of reducing the considered state energies. There are several factors which are responsible for such a behavior, namely, that as long as there is an increment in the hydrostatic pressure, the following happens: (1) the GaAs dielectric constant diminishes, (2) the electron effective mass increases, and (3) the dimensions of the structure decrease. With the increase of the effective mass, both states go down in energies. On the other hand, the reduction of the dielectric constant is related with the reinforcement of the Coulombic interaction and the diminishing of the energies. The reduction of the effective dimensions of the structure will result in a shortening of the effective electron-impurity distance, with the consequence of a decrease in the energies of both states. As it is seen from the figures, the influence of the hydrostatic pressure does not modify the overall phenomenology associated with the energy curves of the donor impurity states.

The effect of an applied electric field on the ground and first excited state energies of the on-ring-center impurity is presented in Figure 8. From the figure, it can be noticed that with the increase of the electric field strength, the energies of the ground and first excited states become reduced. This fact can be explained by the following fact: with the increase of the electric field, the electron cloud is shifted far from the impurity (along −*F*), with a weakening of the electron localization. For this reason, there is a decrease in the energies of both states. It is also apparent that the influence of the electric field on the excited states is greater than on the ground state. This is because the electron ground state is more strongly localized.

In Figure 9, the results for the ground and excited state energies of an on-ring-center donor impurity in a GaAs single QR as a function of its height are depicted. With the increase of the QR’s height, the size quantization weakens which has, as a consequence, the reduction of the energy in each case.

In Table 1, the calculated intraband matrix elements for several configurations of the dimensions of the structure, applied electric field, and hydrostatic pressure are reported.

**Table 1:** Calculated intraband matrix elements

***R***_**1**_	***R***_**2**_	***L***	***F***	***P***	***M***_***if ***_	***M***_***ff ***_	***M***_***ii***_
0	20	20	0	0	0.85	8.34	6.45
6	20	20	0	0	1.54	0.90	10.8
12	20	20	0	0	1.44	10.06	14.50
10	18	20	0	0	1.46	9.80	12.40
10	24	20	0	0	1.63	10.90	15.20
10	30	20	0	0	1.73	12.60	18.00
10	20	5	0	0	1.12	9.11	13.83
10	20	12.5	0	0	1.41	9.55	13.54
10	20	20	0	0	1.76	10.14	12.83
10	20	20	0	0	1.52	9.71	13.30
10	20	20	0	20	1.37	9.41	13.40
10	20	20	0	40	1.22	9.14	13.50
10	20	20	0	0	1.52	9.71	13.30
10	20	20	50	0	1.60	9.84	13.10
10	20	20	100	0	1.76	10.10	12.80

The dimensions of the structure and matrix elements are given in nanometers (nm); electric field, in kilovolts per centimeter (kV/cm); and hydrostatic pressure, in kilobar (kbar).

The linear, nonlinear, and total absorption coefficients for the GaAs-based QR are shown in the Figure 1 as functions of the energy of the incident photon for several values of the ring’s inner radius *R*_1_. The results are for the impurity placed on the QR center. As can be seen from such figure, for the value *R*_1_ = 6 nm, the resonant peak of the absorption is displaced to the region of small photon energies. That is, there is a redshift of the resonant peaks of the intraband optical spectrum. For the value of the inner radius *R*_1_ = 12 nm, the resonant peak of the absorption coefficient is shifted back to the bigger values of the photon energy. In other words, there appears a blueshift of the resonant peaks of the intraband optical spectrum. This phenomenon is due to the fact that the difference of energies between the 1*s* and 2*s* states is larger for *R*_1_ = 12 nm than for the case of *R*_1_ = 6 nm (see Figure 6a). It should be noticed that, with the decrease of the difference of energies between the 1*s* and 2*s* states, the dipole matrix element is larger, and for this reason, the maximum value of the linear and nonlinear absorption coefficients will have an increase.

The effect of the change in the value of the outer ring radius is presented in the Figure 2. There, the variations of the linear, nonlinear, and total absorption coefficients are given as functions of the incident photon energy, with *R*_2_ as a parameter. In the calculations, the impurity is once more considered to be located at the QR center. From Figure 2, it is clear that with the increase of *R*_2_, the resonant peak of the absorption spectrum will become shifted to smaller values of the incident photon energy (redshift). It is also seen that there is a growth of the maximum resonant peak value. In this case, within the whole range of increase of the outer ring, the differences between the 1*s* and 2*s* state energies are progressively reduced (see Figure 6b), and for this reason, there is a redshift. Given the drop in the value of this energy difference, larger values of the dipole matrix element are obtained. So, the maximum values of the linear and nonlinear absorption resonant peaks are bigger.

The effect of the hydrostatic pressure on the linear, nonlinear, and total absorption coefficients is depicted in Figure 3. It is clear that there is an appearance of a hydrostatic pressure-induced blueshift of the resonant peaks of the intraband optical spectrum. This is caused by the increment in the energy difference between the two involved states as a consequence of the increase in the pressure values (see Figure 7). Again, it should be noticed that in this case, the maximum values of the amplitudes of the linear and nonlinear absorption coefficients decrease, which is caused by the decrease of the dipole matrix elements calculated between the mentioned states.

In Figures 4 and 5, the linear, nonlinear, and total absorption coefficients are shown as functions of the energy of the incident photon for several values of the electric field strength and height of the QR, respectively. In both cases, with the increase of electric field strength and height of QR, the localization of the electron is weakened, and as can be seen from Figures 8 and 9, the energy distance between the ground and first excited states decreases. Using this fact, the redshift in the intraband absorption spectrum can be explained straightforwardly.

## Conclusions

In this article, we have studied the combined influence of hydrostatic pressure and in-growth-direction applied electric field on the donor-related linear and nonlinear intraband optical absorption in a GaAs three-dimensional single quantum ring. Our results show that the behavior of the energies of the ground and excited states - and as a consequence, the position of the maximum of the intraband optical absorption related with the transitions from the ground state to the first excited state - strongly depends on the hydrostatic pressure, applied electric field strength, and sizes of the structure. The present results can be useful in understanding the influences of hydrostatic pressure and applied electric field on the impurity states and nonlinear optical properties in single quantum rings.

## Competing interests

The authors declare that they have no competing interests.

## Author’s contributions

MGB, AK, and CAD carried out the numerical work. RLR carried out the analytical work. MEMR carried out the discussion of results. All authors read and approved the final manuscript.
